# Ubiquitin-like modifier FAT10 attenuates RIG-I mediated antiviral signaling by segregating activated RIG-I from its signaling platform

**DOI:** 10.1038/srep23377

**Published:** 2016-03-21

**Authors:** Nhung T.H. Nguyen, Hesung Now, Woo-Jong Kim, Nari Kim, Joo-Yeon Yoo

**Affiliations:** 1 Department of Life Sciences, Pohang University of Science and Technology, Pohang, Korea Hyoja-dong 31, Pohang, 790-784, Republic of Korea

## Abstract

RIG-I is a key cytosolic RNA sensor that mediates innate immune defense against RNA virus. Aberrant RIG-I activity leads to severe pathological states such as autosomal dominant multi-system disorder, inflammatory myophathies and dermatomyositis. Therefore, identification of regulators that ensure efficient defense without harmful immune-pathology is particularly critical to deal with RIG-I-associated diseases. Here, we presented the inflammatory inducible FAT10 as a novel negative regulator of RIG-I-mediated inflammatory response. In various cell lines, FAT10 protein is undetectable unless it is induced by pro-inflammatory cytokines. FAT10 non-covalently associated with the 2CARD domain of RIG-I, and inhibited viral RNA-induced IRF3 and NF-kB activation through modulating the RIG-I protein solubility. We further demonstrated that FAT10 was recruited to RIG-I-TRIM25 to form an inhibitory complex where FAT10 was stabilized by E3 ligase TRIM25. As the result, FAT10 inhibited the antiviral stress granules formation contains RIG-I and sequestered the active RIG-I away from the mitochondria. Our study presented a novel mechanism to dampen RIG-I activity. Highly accumulated FAT10 is observed in various cancers with pro-inflammatory environment, therefore, our finding which uncovered the suppressive effect of the accumulated FAT10 during virus-mediated inflammatory response may also provide molecular clue to understand the carcinogenesis related with infection and inflammation.

Retinoic acid inducible gene I (RIG-I) is a key cytosolic pattern recognition receptor that mediates innate immune responses against RNA virus infection^1^. Upon recognition of intracellular foreign RNAs, RIG-I undergoes a conformational change that exposes its CARD domain for interaction with the mitochondrial antiviral signaling adaptor (MAVS) on the mitochondrial outer membrane^2^. MAVS then triggers the activation of transcription factors IRF3/7 and NF-kB to produce type I interferons (IFNs) and pro-inflammatory cytokines, which are critical mediators of host cellular antiviral response^3^.

Strength and duration of the RIG-I mediated immune-surveillance are tightly regulated to avoid deleterious effects caused by excessive inflammatory response^4^. Although inflammatory cytokines produced during the acute infection is critical for clearing pathogens, healing and regeneration of injured tissues, the sustained or hyper-production of inflammatory cytokines causes auto-immune or inflammatory diseases^5^,^6^. RIG-I hyper-activating mutations have recently been identified in patients with Singleton-Myerten syndrome (SMS), an autosomal dominant multi-system disorder characterized by the presence of glaucoma, psoriasis, and aortic calcification^7^. Altered RIG-I expression has also been linked to inflammatory myophathies pathogenesis and dermatomyositis^8^. Therefore, the identification of regulators that ensure the double-edged function of RIG-I inflammatory response is particularly important to elucidate approaches for the prevention and treatment of RIG-I-associated diseases.

Ubiquitin or ubiquitination is one of the most extensively studied regulators or regulating processes of RIG-I mediated signaling pathway. Multiple ubiquitin E3 ligases, deubiquitinases, and ubiquitin-specific proteases have been identified to particularly modulate the RIG-I-mediated immune response^9^. For example, tripartite motif protein 25 (TRIM25), an E3 ubiquitin/ISG15 ligase^10^, elicits RIG-I activation by inducing K63 poly-ubiquitination^11^. In parallel, although not distinct from ubiquitin, the post-translational modification of cellular targets by ubiquitin-like modifier (UBLs) family also owns great capacity to modulate the protein function. Members of UBLs are recognized as important regulators of RIG-I-mediated signaling in a conjugation dependent manner: SUMO conjugation to RIG-I enhances the IFN production via the augmentation of ubiquitination and interaction with MAVS^12^. Atg5-Atg12 conjugates to RIG-I and MAVS and inhibits the production of type I IFN^13^, while ISG15 conjugates to RIG-I and lowers the stability of RIG-I proteins^14^.

HLA-F adjacent transcription 10 (FAT10), a UBL protein uniquely found in mammals, is well-known as a signal for proteasomal degradation^15^. FAT10 is involved in several cellular processes that occur via either conjugation to target proteins or non-covalent association with other proteins. FAT10 interacts with MAD2 to control chromosome stability and cell cycle progression^16^,^17^, while under proteasome inhibition, it associates with HDAC6 to be sequestered into the aggresome^18^. Highly accumulated FAT10 is observed in various cancers with pro-inflammatory environment^19^,^20^ and emerging evidences implicate FAT10 as a key regulator of inflammatory carcinogenesis^21^,^22^,^23^. However, FAT10 contribution in host-virus interaction, especially in the innate immunity against infection has not been explored.

In the presented manuscript, we demonstrated the inflammatory inducible FAT10 as a novel negative regulator of RIG-I-mediated inflammatory response. Through non-covalent association with an activated form of RIG-I, FAT10 sequesters it away from the signaling transmission platform and attenuates RIG-I mediated antiviral response.

## Results

### Enhanced FAT10 expression inhibits the formation of antiviral stress granules containing RIG-I-G3BP-1

**Figure 1:**
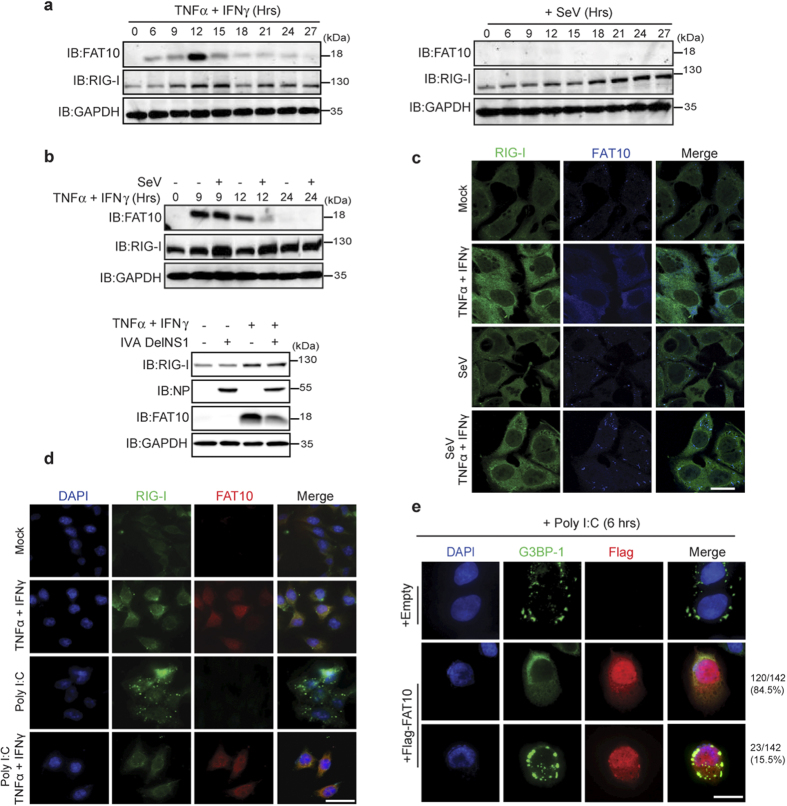
FAT10 inhibits RIG-I containing antiviral stress granules (avSGs) formation. (**a**), HepG2 cells were treated with TNFα and IFNγ (20 ng/ml each) (left) or infected with SeV (right) for indicated time. (**b**), *Top*, After 12 h of SeV infection, TNFα and IFNγ (20 ng/ml each) were treated for indicated time. *Bottom*, After 3 h of IVA DelNS1 infection, TNFα and IFNγ (20 ng/ml each) were treated for 9 h. (**c**), After 3 h of SeV infection, TNFα and IFNγ (20 ng/ml each) were treated for 9 h. Cells were fixed and stained with α-RIG-I and α-FAT10 antibodies. (**d**), HCT116 cells were stimulated with TNFα and IFNγ (20 ng/ml each), poly I:C or together for 9 h. Cells were stained with α-RIG-I and α-FAT10 antibodies. (**e**), HepG2 cells were transfected with Flag-FAT10 or Flag-CMV (empty vector). After 6 h of poly I:C stimulation, cells were fixed and stained with α-Flag and G3BP-1 antibodies. Total 142 FAT10 -transfected cells were examined for G3BP-1 speckle formation.

FAT10, a ubiquitin-like modifier that functions as a regulator of inflammatory carcinogenesis, is transiently induced under TNFα and IFNγ co-stimulation, but not in cells infected with virus alone (Fig. 1a). However, pro-inflammatory cytokine induction of FAT10 expression was severely reduced when virus was infected (Fig. 1b). In virus-infected cells, the level of FAT10 expression by cytokines was significantly reduced, and the concurrent co-localization of FAT10 and RIG-I was observed (Fig. 1c). It has been previously reported that RIG-I is present within the antiviral stress granules (avSGs) along with viral RNA and various antiviral proteins^24^,^25^. Indeed, endogenous RIG-I forms speckles together with GTPase activating protein (SH3 Domain) binding protein 1 (G3BP-1), a stress granule marker protein, in virus-infected cells (Supplementary Fig. 1a). We also have observed that poly I:C induction of RIG-I speckle formation was disrupted by co-treatment with TNFα and IFNγ (Fig. 1d). Furthermore, overexpressed FAT10 specifically blocks the formation of avSGs, which are normally induced by poly I:C stimulation (Fig. 1e). In cells overexpressing Flag-FAT10, G3BP-1-containing avSGs were only detected in 15.5% of cells; the remaining 84.5% of cells displayed no evidence of avSGs formation. In these cells, RIG-I and G3BP-1 were always co-localized (Supplementary Fig. 1b). These results suggest that enhanced FAT10 expression inhibits avSG formation and negatively influences RIG-I signaling pathway.

### FAT10 overexpression inhibits RIG-I-mediated antiviral signaling pathways

**Figure 2:**
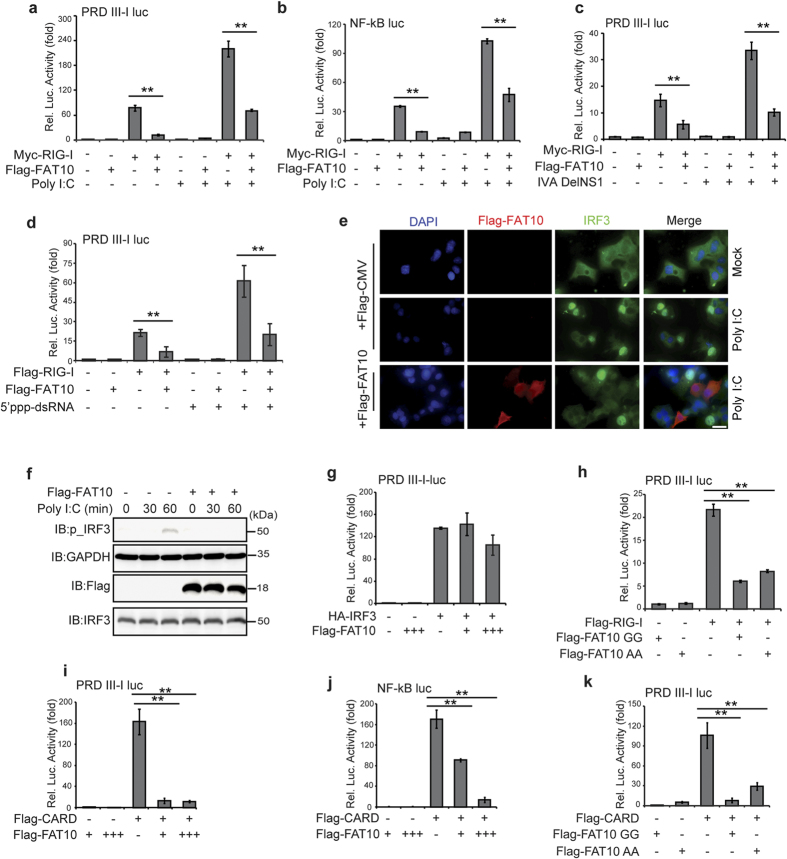
FAT10 overexpression inhibits RIG-I mediated antiviral signaling activities. (**a,b**), HEK293FT cells were co-transfected with indicated plasmid along with PRL-TK and pLuc-PRD III-I or pLuc-NF-kB reporter constructs for 36 h, followed by 12 h of Poly I:C (1 μg/ml) stimulation. Dual luciferase assay and analysis were performed as described. (**c,d**), HEK293FT cells were co-transfected with indicated plasmid along with PRL-TK and pLuc-PRDIII-I reporter constructs for 36 h, followed by IVA DelNS1 infection for 16 h (**c**) or 5′ppp-dsRNA stimulation for 12 h. Dual luciferase assay and analysis were performed as described. (**e**), HepG2 cells were transfected with Flag-FAT10 or Flag-CMV (empty vector) for 36 h, followed by Poly I:C (1 μg/ml) stimulation for 4 h. After fixation, the cells were stained with α-Flag and α-IRF3 antibodies. Representative images of cells in each sample were shown. (**f**), HepG2 cells were transfected with Flag-FAT10 or Flag-CMV (empty vector) for 36 h, followed by Poly I:C (10 μg/ml) stimulation for indicated times. Cells were harvested and phospho-IRF3 level was examined. (**g–k**), HEK293FT cells were transfected with indicated plasmids along with PRL-TK and pLuc-PRD III-I or pLuc NF-kB reporter constructs. Dual luciferase assay and analysis were performed as described. Data presented in graph was from three independent experiments, mean ± SD (error bar). **indicates p < 0.01.

To clarify the effect of enhanced FAT10 expression on RIG-I mediated antiviral signaling pathways, we examined the transactivation activity of the transcription factors interferon regulatory factor-3 (IRF3) and nuclear factor kappa-light-chain-enhancer of activated B cells (NF-kB) using PRD III-I and NF-kB regulatory element reporter assays, respectively. RIG-I-mediated activation of IRF3 and NF-kB was significantly reduced by the overexpression of exogenous Flag-FAT10, both at a basal level and after Poly I:C stimulation (Fig. 2a,b). A similar repressive effect of Flag-FAT10 overexpression on IRF3 activity was observed after viral infection or after stimulation with the RIG-I-specific agonist, 5′ppp-dsRNA (Fig. 2c,d). Consistent with the reporter assay results, both IRF3 nuclear localization and phosphorylation of IRF3 were blocked by FAT10 overexpression (Fig. 2e,f). In contrast, overexpressed FAT10 did not alter increased PRD III-I activity following IRF3 overexpression (Fig. 2g), suggesting that FAT10 specifically acts upstream of IRF3 activation to attenuate RIG-I-mediated signaling pathways.

FAT10 can exert its effects through both conjugation-dependent and conjugation-independent mechanisms^26^. Using a conjugation-defective FAT10 mutant, in which an AA was substituted for GG at the FAT10 C-terminus, we found that covalent-conjugation of FAT10 is not required for its inhibitory effect on RIG-I mediated antiviral responses (Fig. 2h).

Next, we investigated whether constitutive RIG-I activity could be inhibited by FAT10 overexpression. An expression vector encoding the tandem caspase activation and recruitment domains (2CARD) of RIG-I was transfected into cells and the trans-activation activities of both IRF3 and NF-kB were measured without further stimulation. The results showed that 2CARD-RIG-I constitutive activation was significantly reduced by Flag-FAT10 overexpression (Fig. 2i,j). This inhibitory effect of FAT10 on constitutive RIG-I activity was also independent of protein conjugation (Fig. 2k). These findings confirm that FAT10 negatively regulates the RIG-I-mediated anti-viral signaling pathway.

### Identification of the FAT10 protein as a novel RIG-I interacting protein

**Figure 3:**
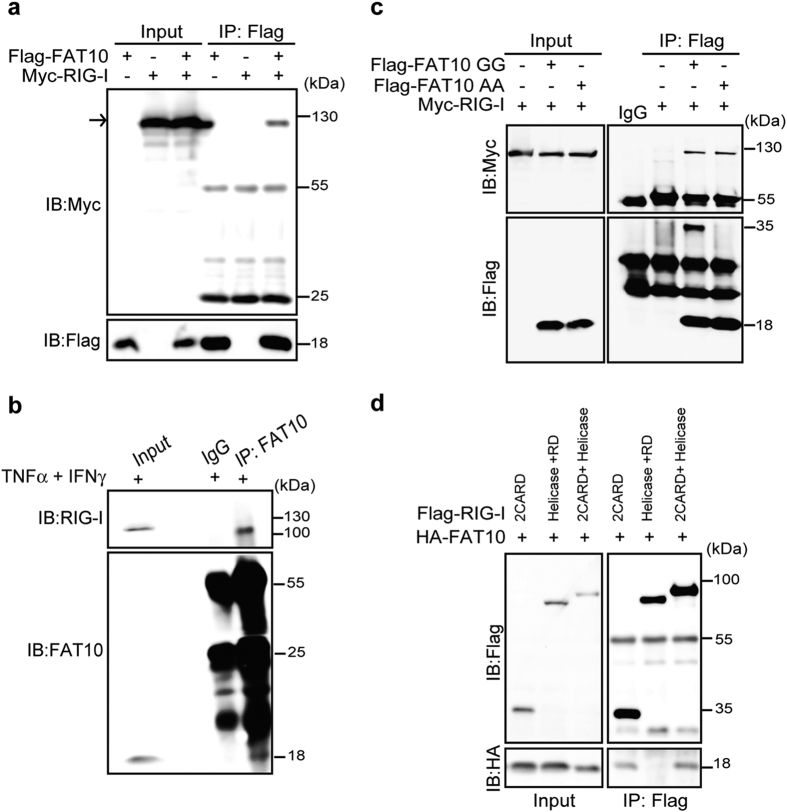
FAT10 physically interacts with RIG-I. (**a**), The physical interaction of RIG-I and FAT10 was examined using α-Flag immunoprecipitation of total lysate from Myc-RIG-I and Flag-FAT10 transfected HEK293FT cells. (**b**), HepG2 cells were treated with TNFα and IFNγ (50 ng/ml each) for 12 h. Total lysates were immunoprecipitated with α-FAT10 antibody. (**c**), HEK293FT cells were transfected with Myc-RIG-I and Flag-FAT10 GG or Flag-FAT10 AA for 48 h, and physical interaction was examined by immunoprecipitation of total lysates with α-Flag antibody. (**d**), HEK293FT cells were transfected with truncated forms of Flag-RIG-I along with HA-FAT10 for 48 h, followed by the immunoprecipitation of total lysates with α-Flag antibody. Bands at 55 kDa and 25 kDa are heavy and light chains of IgG.

Based on the co-localization of RIG-I and FAT10, we evaluated whether FAT10 regulates RIG-I protein via protein-protein interaction by investigating exogenous Flag-FAT10 and Myc-RIG-I physical interactions following transfection into HEK293FT cells (Fig. 3a). Immunoprecipitation using α-Flag antibody clearly detected overexpressed Myc-RIG-I, confirming the physical interaction of FAT10 and RIG-I proteins. Those proteins that were pulled down together with FAT10 were not FAT10-conjugated forms of RIG-I because their molecular weights were similar to those of the free forms of Myc-RIG-I observed in SDS-containing gels. Physical interaction between endogenous FAT10 and RIG-I proteins was also observed in the lysates from cells stimulated with TNFα and IFNγ (Fig. 3b). Consistent with the effect of FAT10 on RIG-I-mediated intracellular signaling, the conjugation-defective FAT10 AA protein also interacted with RIG-I, confirming that FAT10 interaction with RIG-I is independent of its conjugation ability (Fig. 3c). Next, using a series of deletion construct, we mapped the RIG-I domain responsible for FAT10 interaction (Fig. 3d). RIG-I contains various functional domain, including 2CARD domains, Helicase domain and Repressor Domain (RD). Therefore various forms of truncated mutants, which contains only 2CARD domain, lacks 2CARD domain, or lacks RD domain were generated. When the RIG-I N-terminal 2 CARD domain was deleted, its interaction with FAT10 was abolished, whereas the 2CARD domain of RIG-I was sufficient to maintain their interaction (Fig. 3d). This finding is consistent with a previous observation that IRF3 activation induced by 2CARD overexpression was inhibited by FAT10 overexpression (Fig. 2k). Based on these data, we concluded that FAT10 associates non-covalently with RIG-I and that the RIG-I N-terminal 2CARD domain is essential for this interaction.

### FAT10 overexpression leads to 2CARD-RIG-I precipitation as an insoluble aggregate

**Figure 4:**
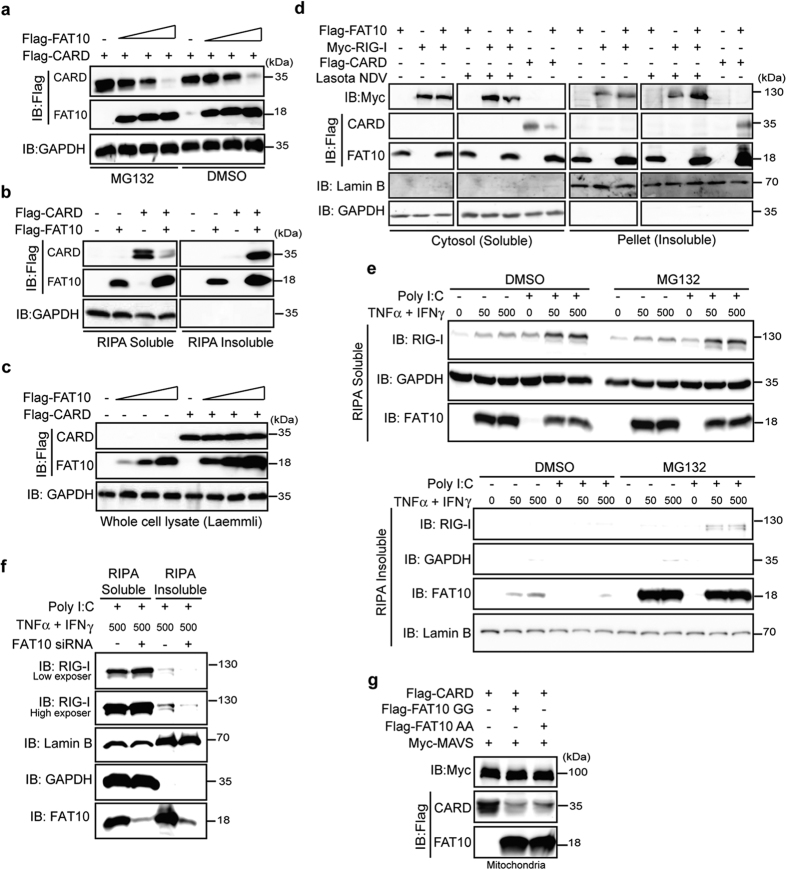
Overexpressed FAT10 induces activated RIG-I (2CARD) precipitation. (**a**), HEK293FT cells were transfected with indicated plasmids for 48 h. Cells were treated with 20 μM MG132. (**b**), HEK293FT cells were transfected with indicated plasmids for 48 h. After harvesting, the soluble and insoluble fractions were prepared using RIPA lysis buffer. The protein levels of FAT10 and CARD in each fraction were analyzed. (**c**), HEK293FT cells were transfected with indicated plasmids for 48 h, whole cell lysates were prepared in Laemmli buffer containing 5% β-mercaptoethanol. (**d**), HEK293FT cells were transfected with indicated plasmids for 48 h, Lasota NDV was infected for 12 h before harvest. Cytosol (soluble) and insoluble fractions were prepared using sucrose buffer. Protein levels of FAT10 and RIG-I in each fraction were analyzed. (**e**), HepG2 cells were pre-treated with TNFα and IFNγ with final concentration (ng/ml) as indicated for 2 h following by Poly I:C (10 μg/ml) transfection. MG132 (20 μM) or DMSO was treated for 4 h before harvesting. Endogenous RIG-I and FAT10 protein levels were analyzed in the RIPA soluble and insoluble fractions. (**f**), same as e, except HepG2 cells were transfected with control or FAT10 siRNA. (**g**), HEK293FT cells were transfected with indicated plasmids, and the protein levels of FAT10 and CARD were examined in the mitochondria enriched fraction.

When a 2CARD-RIG-I protein was co-expressed with FAT10, we repeatedly observed that FAT10 decreased 2CARD-RIG-I protein levels in a dose-dependent manner (Fig. 4a). Further, we investigated whether overexpressed FAT10 alters 2CARD-RIG-I protein stability. However, treating cells with MG132 or chloroquine, which specifically block either classical 26S proteasome- or lysosome-dependent protein degradation pathways, respectively, did not perturb the effect of overexpressed FAT10 on 2CARD-RIG-I protein concentration (Fig. 4a, Supplementary Fig. 2a).

Because it has been previously reported that FAT10 is found in the aggresomes^18^ and that it tends to precipitate in an insoluble form^27^, we investigated whether FAT10 directs RIG-I into an insoluble fraction via protein-protein interaction. To test whether overexpressed FAT10 alters the solubility of 2CARD-RIG-I proteins, we harvested whole cell lysates and separated the RIPA-soluble and -insoluble fractions. In every case, increasing FAT10 expression decreased the soluble 2CARD-RIG-I protein present and simultaneously increased the concentration of FAT10 and 2CARD-RIG-I in the insoluble fraction (Fig. 4b, Supplementary Fig. 2b). However, in whole cell lysates prepared in the Lammeli buffer, the 2CARD-RIG-I total protein concentration remained unchanged despite increasing levels of FAT10 overexpression (Fig. 4c). These results indicate that FAT10 did not alter 2CARD-RIG-I protein stability but instead simply relocated 2CARD-RIG-I from the soluble to insoluble fraction. Because the FAT10 overexpression suppressed 2CARD-RIG-I activity in parallel with its relocation into the insoluble fraction, we concluded that FAT10 inhibits 2CARD-RIG-I activity by sequestering the protein in the insoluble cell fraction.

Next, we examined whether FAT10 also regulates the solubility of full length RIG-I proteins. When Myc-RIG-I was co-overexpressed with FAT10, changes in cytosolic RIG-I protein levels were barely detectable under basal conditions. However, viral infection decreased amount of RIG-I protein present in the cytosol fraction and at the same time, increased the level of RIG-I in the insoluble fraction (Fig. 4d). Next, we examined whether changes in endogenous RIG-I solubility occurred. To cause excessive amounts of endogenous FAT10 accumulation, FAT10 production was induced by exposure to high doses of TNFα and IFNγ and protein degradation was blocked by the 26S proteasome inhibitor MG132. Under these harsh conditions, endogenous RIG-I was observed in the insoluble fraction along with the FAT10 proteins (Fig. 4e). Furthermore, the amounts of insoluble RIG-I diminished when endogenous FAT10 was silenced (Fig. 4f). These data indicate that FAT10 sequesters the active form of RIG-I into insoluble precipitate, only when excessive amounts of FAT10 are present.

Because activated RIG-I undergoes a conformational change that facilitates its interaction with MAVS on the mitochondrial outer membrane, we determined whether altered RIG-I protein solubility influenced the translocation of active RIG-I into the mitochondria. Cells were co-transfected with both 2CARD-RIG-I and FAT10, and mitochondrial concentrations of FAT10 and 2CARD-RIG-I were measured (Supplementary Fig. 2c,d). Results indicated that most of 2CARD-RIG-I protein detected along with mitochondrial MAVS disappeared when FAT10 was overexpressed. Furthermore, the overexpression of both FAT10-GG and FAT10-AA had a similar effect on 2CARD-RIG-I localization to the mitochondria (Fig. 4g), indicating that FAT10 moves activated RIG-I into the insoluble cellular compartment and prevents RIG-I from targeting the mitochondria in a conjugation-independent manner.

### The T55 residue in the RIG-I CARD domain is required for the interaction with FAT10

**Figure 5:**
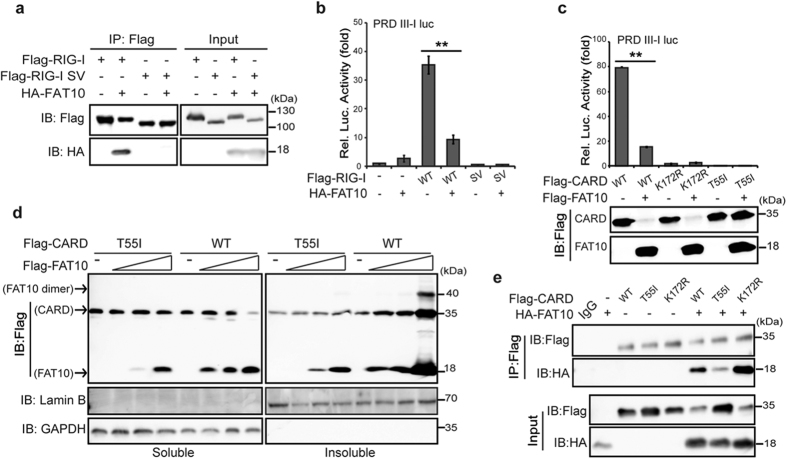
T55 residue of RIG-I is needed to mediate interaction with FAT10. (**a**), HEK293FT cells were co-transfected as indicated for 48 h, following by immunoprecipitation with α-Flag antibody. (**b,c**), HEK293FT cells were co-transfected with indicated plasmid along with PRL-TK and pLuc-PRD III-I reporter constructs for 48 h. Dual luciferase assay and analysis were performed as described. (**d**), HEK293FT cells were transfected with an increasing dose of Flag-FAT10 and indicated plasmids for 48 h. After harvesting, soluble and insoluble fractions were prepared in the passive lysis buffer. The protein levels of FAT10 and CARD WT or mutant in each fraction were analyzed using the indicated antibody. (**e**), The physical interaction of CARD mutants and FAT10 were examined using immunoprecipitation of total lysate from transfected HEK293FT cells with α-Flag antibody.

The sequestering effect of FAT10 on RIG-I was inevitable when the constitutive active 2CARD-RIG-I was used or when RIG-I was activated by viral infection. Furthermore, a natural RIG-I splicing variant (RIG-I SV) that harbors a short deletion within the first CARD domain failed to interact with FAT10 (Fig. 5a). RIG-I SV lost its ability to interact with the E3 ligase TRIM25, and as the results demonstrated, it could not initiate downstream signaling^28^ (Fig. 5b). Therefore, to test whether active RIG-I is a selective target of FAT10 regulation, we introduced inactivating point mutations into 2CARD-RIG-I. A T55I mutation abolishes RIG-I interaction with TRIM25, whereas a K172R mutation results in the loss of K63 ubiquitination^11^,^28^. These generated mutants were inactive as measured by an IRF3 activation luciferase assay (Fig. 5c, upper). We also examined the presence of 2CARD-RIG-I and FAT10 in the soluble fraction by using lysates of the same luciferase assay (Fig. 5c, bottom). When FAT10 was co-expressed, both the normal and K172R mutant proteins disappeared from the soluble fraction; however, the T55I mutant protein remained. Indeed, the solubility of the T55I mutant protein did not change, even in the presence of high concentrations of FAT10 (Fig. 5d). Moreover, we examined whether changes in RIG-I solubility are dependent upon FAT10 interaction. In agreement with the observed changes in solubility, the interaction between T55I and FAT10 was much weaker compared with WT or K172R interactions (Fig. 5e). These results indicate that K63 ubiquitination of the K172 residue is not required, but that an intact RIG-I-T55 residue is required for FAT10 interaction. This interaction appeared to be a pre-requisite for FAT10-mediated RIG-I precipitation.

### TRIM25 stabilizes the FAT10 protein by inhibiting its degradation

**Figure 6:**
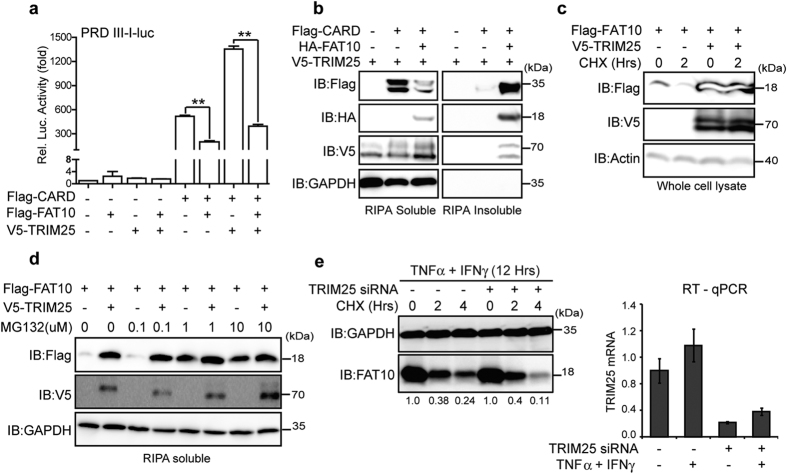
TRIM25 stabilizes FAT10 protein by inhibition of proteasomal degradation. (**a**), HEK293FT cells were co-transfected with indicated plasmid along with PRL-TK and pLuc-PRD III-I reporter constructs for 48 h. Dual luciferase assay was performed as described. (**b**), HEK293FT cells were transfected with indicated plasmids for 48 h. After harvesting, soluble and insoluble fraction was prepared as described in RIPA lysis buffer. The protein levels of FAT10, TRIM25, and CARD in each fraction were analyzed using indicated antibodies. (**c**), HEK293FT cells were transfected with indicated plasmids for 36 h. After CHX (50 μg/ml) treatment for 2 h, whole cell lysate was prepared in Laemmeli buffer. (**d**), HEK293FT cells were transfected with indicated plasmids for 36 h, and then treated with an indicated dose of MG132 for 12 h. After harvesting, soluble and insoluble fractions were prepared in RIPA lysis buffer. (**e**), HepG2 cells were transfected with TRIM25 siRNA or control siRNA for 36 h, and treated with TNFα and IFNγ for additional 12 h. After CHX (50 μg/ml) treatment for indicated time, whole cell lysate was prepared in Laemmeli buffer. Endogenous FAT10 level was examined (left) and silencing of TRIM25 by siRNA was confirmed by qPCR (right). Band intensity of FAT10 was normalized with GAPDH (left).

**Figure 7:**
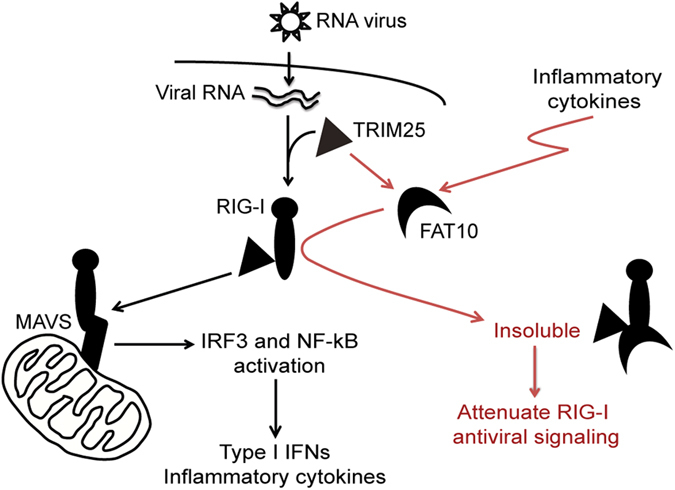
Proposed working mechanism. Upon RNA virus infection, TRIM25 mediated K63 ubiquitination of RIG-I is essential for RIG-I targeting to MAVS in mitochondria and transmitting downstream signaling. As a consequence, type I IFNs and inflammatory cytokines are produced. Under inflammatory conditions, FAT10 protein is accumulated. FAT10 interacts with the CARD domain of RIG-I and leads to RIG-I precipitation in the insoluble form. FAT10 is not as stable as it is degraded via proteasome, but TRIM25 can stabilize FAT10 and assist its accumulation. Therefore, FAT10 selectively blocks active RIG-I targeting to its signaling platform (mitochondria) and efficiently attenuates RIG-I inflammatory response.

Because the RIG-I T55 residue is essential for interaction with the TRIM25 E3 ligase, it is possible that FAT10 competes with TRIM25 for 2CARD-RIG-I binding, thereby inhibiting TRIM25-mediated RIG-I activation. Consequently, we examined whether FAT10 overexpression suppressed TRIM25-mediated RIG-I signaling activity (Fig. 6a). 2CARD-RIG-I mediated IRF3 activation was further enhanced by TRIM25 over-expression, and this enhanced activity was significantly reduced by FAT10 overexpression. In contrast, TRIM25 overexpression slightly altered the inhibitory effect of FAT10 on 2CARD-RIG-I. Moreover, TRIM25 overexpression did not prevent 2CARD-RIG-I precipitation by FAT10 (Fig. 6b). In addition, transfected exogenous Flag-FAT10 protein levels were substantially enhanced by TRIM25 co-expression (Fig. 6c). FAT10 is an unstable protein with a half-life of 1 h, with its protein stability regulated through 26S proteasome-dependent degradation^15^. Interestingly, FAT10 stability was significantly enhanced by TRIM25 overexpression (Fig. 6c). After 2 h of treatment with cycloheximide, most of the Flag-FAT10 protein had disappeared. However, when TRIM25 was present, there was no significant decrease in Flag-FAT10 protein even after cycloheximide treatment. Co-transfection with V5-TRIM25 resulted in an effect that was similar to that with MG132 (Fig. 6d). Furthermore, when TRIM25 was silenced, endogenous FAT10 protein stability was reduced (Fig. 6e). Collectively, these data suggest that TRIM25 stabilizes FAT10. Of note, TRIM25 was recently reported to enhance p53 stability through the inhibition of proteasomal p53 protein degradation^29^. We propose that FAT10 is recruited to RIG-I-TRIM25 to form an inhibitory complex where FAT10 is stabilized. As a result, stabilized FAT10 sequesters active RIG-I into an insoluble fraction that restricts RIG-I localization to MAVS containing mitochondria (Fig. 7).

## Discussion

Our study has demonstrated how the coordinated interplay of RIG-I, TRIM25, and FAT10 regulate the antiviral innate inflammatory response. We have discovered a dual role of TRIM25 in the regulation of RIG-I activity. When a host cell detects a viral infection, TRIM25 induces unanchored K63 ubiquitination of RIG-I, thereby activating RIG-I antiviral defense responses. Upon the accumulation of the proinflammatory-inducible protein FAT10, RIG-I activity is suppressed. Because the basal FAT10 level is low and silencing basal FAT10 expression did not considerably affect Poly I:C induced avSGs formation (Supplementary Fig. 3a), we concluded that FAT10 may elicit its repressive function mainly under inflammation conditions. TRIM25 also enhanced FAT10 stability, thereby strengthening the inhibitory effect of FAT10 to attenuate RIG-I-mediated inflammatory response, most likely in situations of severe inflammation.

Because of their importance in the recognition and regulation of viral infection, RLR-mediated signaling activities are tightly controlled by various mechanisms. For example, NLRX1 regulates RIG-I targeting to MAVS within the mitochondria^30^, NLRX5 and HSCARG regulate RIG-I downstream signal transduction^31^,^32^, and ubiquitin and ISG15 regulate RIG-I protein concentration through K48 polyubiquitination and ISGylation^9^,^14^. Here we have presented a novel pathway for regulating RIG-I signaling activity involving altered protein solubility. It has been previously reported that RIG-I is found in the insoluble fraction in the HIV-1-infected monocyte-derived macrophages^33^. Furthermore, the NS proteins of SFTSVs virus have been recently shown to inhibit IFN signaling through the active relocation of RLR signaling components, including the movement of RIG-I, TBK1, and TRIM25 to an insoluble fraction, which was described as an NS-induced cytoplasmic structure^34^. Therefore, the redistribution of RIG-I and related components may be employed by viruses as a way to overcome the host anti-viral response.

To date, the physiological functions of FAT10 in the innate immune response to viral infection have not been reported, although its potential involvement has been generally implicated. FAT10 expression is up regulated during Kaposi sarcoma-associated herpesvirus infection^35^ and in HIV-associated neuropathy^36^. FAT10 interacts with the HIV accessory protein Vpr to mediate Vpr-induced cell death^36^. In addition, FAT10 functions in the intracellular defense against bacteria by decorating cytoplasmic *Salmonella Typhimurium* that has been targeted for xenophagy^37^. Furthermore, FAT10-deficient mice are more sensitive to endotoxin challenge^38^, and FAT10 deletion in rats negatively affects virus-triggered autoimmune diabetes^39^, suggesting potential regulatory role of FAT10 during infection. The present study is the first to demonstrate this previously unreported function of FAT10 in an antiviral response. Our findings highlight the importance of controlling FAT10 to efficiently handle the various pathologies caused by RNA viruses. Through its ability to inhibit RIG-I, an important sensor for innate immunity, FAT10 ensures the precise regulation of the virus- mediated inflammatory response. However, during chronic infection and inflammation, the suppressive effect of accumulated FAT10 might also contribute to the successful establishment of a persistent infection that initiates an infection-triggered cancer.

FAT10 was previously reported to be present in the insoluble fraction^27^, and has also been reported to conjugate with polyglutamine proteins and control their solubility^40^. Moreover, an active LRRFIP2 adaptor protein has been reported to translocate from the membrane to the insoluble fraction in a manner that requires FAT10 conjugation^41^. Herein, we have demonstrated that overexpressed FAT10 can control protein solubility through the non-covalent protein-protein interaction. Endogenous FAT10 was also found in the insoluble fraction, with the protein concentration in the insoluble fraction further increased by the inhibition of proteasomal degradation. Endogenous RIG-I was also observed in the insoluble fraction in FAT10 dependent manner (Fig. 4e,f), suggesting that the insoluble RIG-I and FAT10 proteins were targeted for degradation via the proteasome. The detergent-insoluble cell fraction concentrates chromatin, cellular structures, aggregated proteins, and cytoskeleton-associated proteins^40^,^42^,^43^,^44^,^45^. Transition from the soluble to the insoluble fraction could mean changes in cellular localization, protein conformation, or protein association. Proteins can be found in the insoluble fraction under specific physiological conditions, such as pathological transition or infection as a result of changes in their biochemical properties^44^,^46^,^47^. A change in RIG-I protein solubility may require additional post-translational modifications, although this possibility remains to be investigated.

The current study and previously published reports have shown that FAT10 is both inducible and extremely unstable. As a result, the FAT10 protein is barely detectable in most normal, untreated cells, and it is only transiently induced by inflammatory signaling^17^. We found that TRIM25 stabilizes FAT10 through the inhibition of its proteasome-dependent degradation. Recently, TRIM25 has been shown to enhance stability of p53 and mdm2^29^, and TRIM25 overexpression in ovarian tumors has been reported^48^. Our finding that additional TRIM25 activity controls the stability of short-lived proteins may explain how the FAT10 protein accumulates to a high level in various cancers. In conjunction with its ability to counteract p53, Akt, β-catenin^21^,^22^,^23^ and chromatin stability^17^, the suppressive function of accumulated FAT10 may provide a molecular clue to help understand the associations between carcinogenesis, infection and inflammation.

## Materials and Methods

### Plasmids

Flag-RIG-I plasmid was provided by Dr. Takashi Fujita (Institute for Virus Research, Kyoto University) and Myc-RIG-I was made from this construct by replacing Flag tag by Myc tag. The construction of Flag-RIG-I SV (an alternatively spliced variant of RIG-I), Flag-Helicase + RD, Flag-CARD, Flag-CARD + Helicase were described previously^49^,^50^. To generate Flag-CARD T55I and K172R, appropriate point mutations were introduced into Flag-CARD using the QuickChange site-directed mutagenesis kit (Stratagene). Plasmid information for Flag-FAT10 GG and its non-conjugating mutant Flag-FAT10 AA has been previously described^21^. HA-FAT10 was provided by Dr. Groettrup Marcus (University of Konstanz, Germany). V5-TRIM25 was given by Dr. Jae U. Jung (University of Southern Califonia, USA). The pLuc-NF-kB was purchased from Clonetech and the pLuc-PRD III-I was given by Dr. Katherine A. Fitzgerald (University of Massachusetts, USA). pSG5-IRF3-HA was given by Dr. Jae Myun Lee (Yonsei University, Korea). From pcDNA3-Flag-MAVS (kindly given by Dr. Chen, Howard Hughes Medical Institute, USA), myc-MAVS was constructed by cloning MAVS into PCS2 + MT-myc empty vector. siRNAs targeting human TRIM25 and human FAT10 (Supplementary Table S1) were synthesized by GenePharm (Shanghai, China).

### Cell culture, transfection and stimulation

HepG2 (American Type Culture Collection, Manassas, VA), HCT116 (provided by Dr. Bert Vogelstein, Johns Hopkins University, Baltimore, MD) and human embryonic kidney HEK293FT (Life Technologies) were cultured under the recommended conditions. HepG2 cells were maintained in 10% FBS (Hyclone) and 1% penicillin/streptomycin (Invitrogen) containing MEM media. HEK293FT and HCT116 cells were maintained in 10% FBS and 1% penicillin/streptomycin containing DMEM media. For transient transfection in HEK293FT cells, equal quantities of expression plasmids were transfected by calcium phosphate method. For other cells, plasmids were transfected by Lipofectamine 2000 (Invitrogene) according to the manufacturer’s instruction. For agonists stimulation, indicated concentration of Poly I:C (Amersham Biosciences) or 5′ppp-dsRNA (Invivogen) was transfected using Lipofectamine 2000. For cytokines stimulation, if not specifically mentioned, 20 ng/ml TNFα or 20 ng/ml IFNγ (R&D systems, Minneapolis, MN) were applied to cells for indicated time. MG132, Chloroquine (Cq), and Cycloheximide (CHX) were purchased from Sigma (MO, US) and treated to cells as indicated. For knock down of target gene, siRNA were transfected into the cells using Lipofectamine 2000 (Invitrogene) according to the instruction of manufacturer.

### Virus infection

IVA DelNS1 (Influenza A/PR/8/34, ΔNS1) virus was provided by Dr. Huan Huu Nguyen (National Vaccine Institute, Seoul, Korea). Newcastle disease virus (NDV, LaSota strain) was provided by National Veterinary Research Quarantine Service, South Korea and Sendai virus (SeV) was provided by Dr. P. Palese (Mount Sinai School of Medicine, USA). For virus infection, cells were washed with 1X PBS before treatment with culture medium (mock) or indicated virus in serum-free and antibiotic free medium. After absorption at 37 °C for 1 h, media was changed, and cells were cultured for various periods in the serum-containing media. Mock indicated untreated sample.

### RNA isolation and real-time quantitative RT-PCR

RNA was isolated with the RNAiso reagent (Takara, Shiga, Japan) according to the instructions of manufacturer. Total RNA (1μg) was reverse transcribed using the ImProm-II TM Reverse Transcription Kit (Promega) with Oligo dT as primer. Quantitative real-time PCR was performed with StepOne plus (Applied Biosystems) using SYBR-Premix Ex Taq (Takara). Primer information was provided (Table 1).

### Immunobloting and antibodies

For immunoblotting, protein samples were separated on 12% SDS-PAGE and transferred to nitrocellulose membrane (Whatman, NJ). Membranes were incubated with 7% skim milk (in PBST 1X), followed by antibody hybridization. α-Tubulin, α-Myc, α-GFP, α-HA and goat α-RIG-I antibodies were purchased from Santa Cruz Biotech. α-Mitofilin was purchased from NOVUS. α-Flag and α-V5 were purchased from Sigma and Invitrogen respectively. α-IRF3, α-RIG-I and α-FAT10 were purchased from Enzo Life Science, α-phospho IRF3 (Ser369), α-G3BP-1 was purchased from Cell Signaling. α-NP antibody was purchased from Abcam. Immunoreactive signals were detected by enhanced chemiluminescence in horseradish peroxidase (Pierce Biotechnology, MA). For immunofluorescence experiments: Alexa 488-, 568-, 647- conjugated anti-mouse, anti-rabbit, anti-goat IgG antibody purchased from Invitrogen were used as secondary antibodies.

### Immunoprecipitation

Cells were harvested in IP lysis buffer (50 mM Tris-HCl pH 8.0, 150 mM NaCl, 1% NP40, 0.5% deoxycholic acid, 0.1% SDS and 1 mM EDTA pH 8.0) with protease inhibitors (1 mM DTT, 0.5 mM PMSF, 5 μg/ml leupeptin, 2 μg/ml pepstatin A, 5 μg/ml aprotin and 1 mM benzamindine). Total cellular lysates (1mg) were pre-cleared with mouse immunoglobulin G (IgG) and protein G/protein A - agarose beads (Calbiochem, La Jolla, CA) and then incubated with indicated antibodies (2 μg/mg of lysates) or mouse IgG overnight at 4 °C. After multiple 4 rounds of washing in 1 ml of IP lysis buffer, proteins were eluted by boiling at 95 °C in laemmli buffer (Biophoretic) containing 5% β-mercaptoethanol (Sigma-Aldrich). For endogenous IP, β-mercaptoethanol was not added in laemmli buffer. Proteins were analyzed on SDS-polyacrylamide gels. Input controls consisted of 5% total cell lysates.

### Luciferase reporter assay

Cells were cotransfected with pLuc-NF-kB or pLuc-PRD III-I reporter construct together with pRL-TK, along with indicated plasmids. Dual luciferase reporter assay was performed according to the manufacturer’s instruction (Promega). Firefly luciferase activity was measured and normalized with the value of renilla activity. “Rel. Luc. Activity (Fold)” relative value to control sample (unstimulated, transfected with empty vector and luciferase plasmids) was used. Data obtained from three independent experiments, mean ± SD. **indicates p < 0.01.

### Immunofluorescence microscopy

Cells were fixed with 4% paraformaldehyde for 10 min, permeabilized with 0.2% Triton X100 in PBS for 8 min at RT, blocked with 1% goat serum in PBST for 1 h at RT, and incubated at 4 °C overnight with the relevant primary antibodies. After incubation with secondary antibodies at RT for 1 hour, nuclei were stained with Hoechst 33258 (Sigma). Slides were mounted and then images were acquired using fluorescence microscopy (Carl Zeiss, Jena, Germany) equipped with an ApoTome (Carl Zeiss) through a 63X oil fluorescence objective. In Supplementary Fig. 1b, images were acquired using a modified Zeiss Axiovert 200M microscope equipped with a Photometrics Coolsnap HQ camera through a 40X objective. The scale bar represents 10 μm.

### Confocal microscopy and avSGs quantification

In Fig. 1c and Supplementary Fig. 3a, images were acquired using a Zeiss LSM 510 META confocal laser scanning microscopy through a 63X oil objective. The images were processed using the LSM 5 image browser and presented as pseudo images. The scale bar represents 10 μm. % cells formed stress granules in each sample were calculated in 10 random fields (each contains 8–12 cells). Data obtained from three independent samples, mean ± SD.

### Subcellular fractionation

We used three different kinds of cell lysis buffer to harvest the soluble and insoluble fraction separately. For Passive lysis buffer (Promega) or RIPA lysis buffer (50 mM Tris-HCl pH 7.4, 150 mM NaCl, 1% NP40, 0.5% deoxycholic acid, 0.1% SDS) supplemented with protease inhibitors, cellular lysates were collected and centrifuged at 12,000 *g,* 30 min. The supernatant was collected as soluble fraction and remaining pellet was boiled at 95 °C in laemmli buffer containing 5% β-mercaptoethanol for 10 min, as insoluble fraction. For Sucrose buffer (320 nM sucrose, 10 mM Tris-Cl pH 8.0, 3 mM CaCl_2_, 2 mM MgOAc, 0.1 mM EDTA, 0.5% NP-40, with protease inhibitors), cells lysates were harvested after centrifugation at 600 *g*. The cytosol (supernatant) was collected, and the pellet was washed with sucrose buffer without NP40 and boiled at 95 °C in laemmli buffer containing 5% β-mercaptoethanol to prepare insoluble fraction.

### Mitochondrial/cytosol fractionation

Harvested cells were washed in 1X PBS, suspended in a mitochondria isolation buffer (250 mM sucrose, 1 mM EGTA, 1 mM MgCl2, 0.5 mM DTT, 10 mM Tris-Cl pH 8.0, EDTA-free protease inhibitors), and disrupted by dounce homogenization. The homogenate was centrifuged at 800 *g* for 10 min. The supernatant was harvested and centrifugated 8,000 *g* for 10 min. The resulting pellet was collected as mitochondria fraction while the supernatant (cytosolic fraction) was cleared by further centrifugation for 30 min at 12,000 *g* before harvesting. GAPDH was used as the marker of the cytosolic fraction and Mitofilin was used as the marker of the mitochondrial fraction.

### Statistical Analysis

Statistical analyses were performed by two-tailed Student’s t test. P < 0.05 was considered statistically significantly different.

## Additional Information

**How to cite this article**: Nguyen, N.T.H. *et al.* Ubiquitin-like modifier FAT10 attenuates RIG-I mediated antiviral signaling by segregating activated RIG-I from its signaling platform. *Sci. Rep.* **6**, 23377; doi: 10.1038/srep23377 (2016).

## Supplementary Material

Supplementary Information
