# Supplementary material for: Ubiquitin-like modifier FAT10 attenuates RIG-I mediated antiviral signaling by segregating activated RIG-I from its signaling platform
Source: Sci Rep. 2016 Mar 21;6:23377. doi: 10.1038/srep23377 (PMC4800306; doi:10.1038/srep23377)
Supplement: Supplementary Information [file srep23377-s1.pdf]

Supplementary information

Ubiquitin-like modifier FAT10 attenuates RIG-I mediated antiviral signaling by segregating activated RIG-I from its signaling platform

Authors

Nhung Thi Hong Nguyen[1], Hesung Now[1], Woo-Jong Kim, Nari Kim, Joo-Yeon Yoo[*]

Supplementary Figure 1

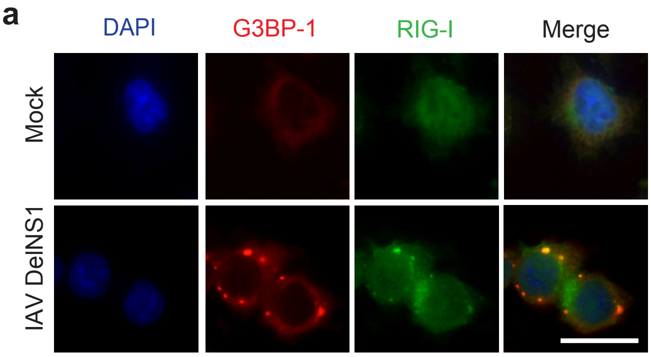

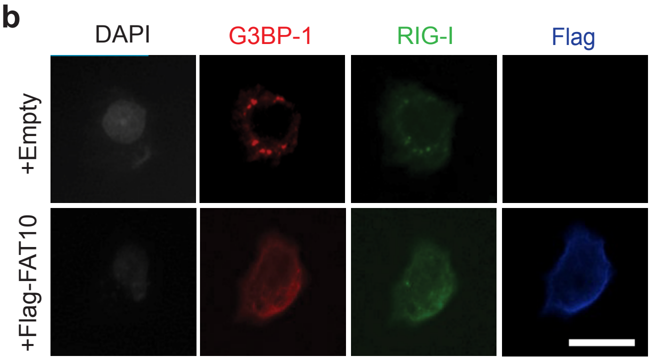

Supplementary Fig. 2

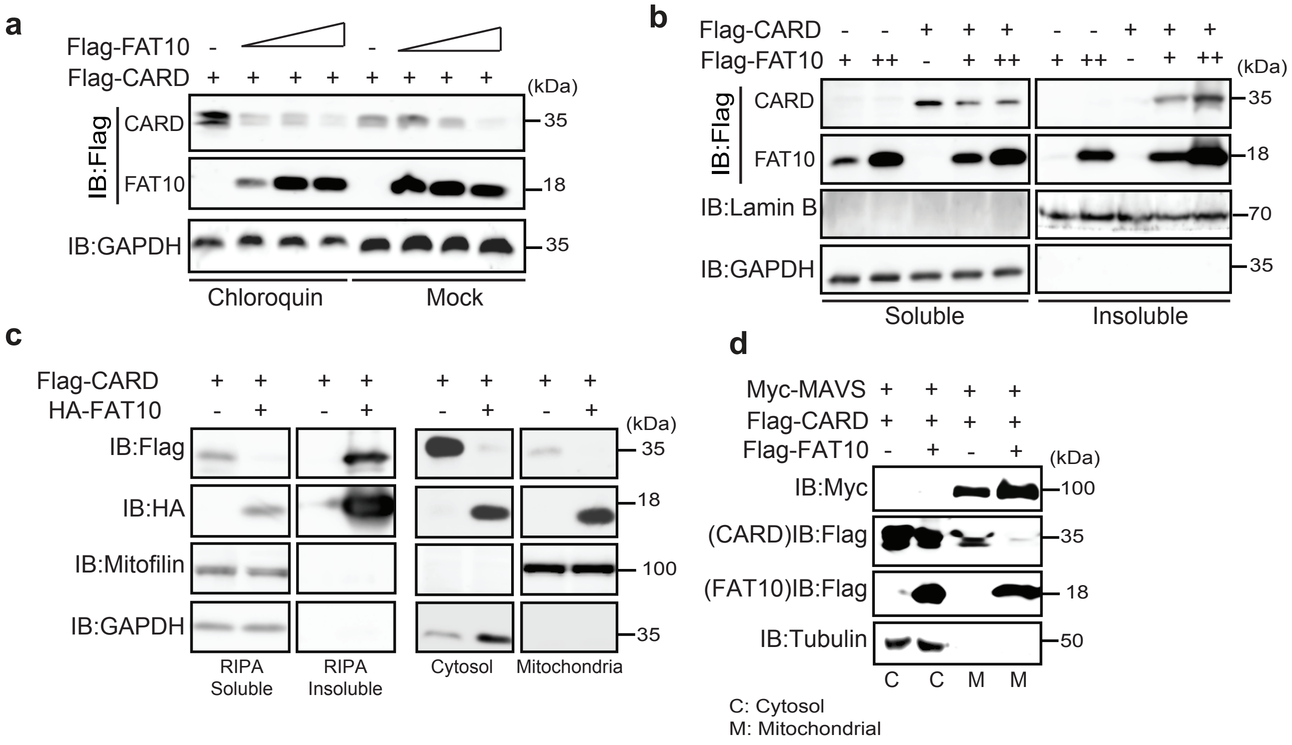

Supplementary Fig. 3

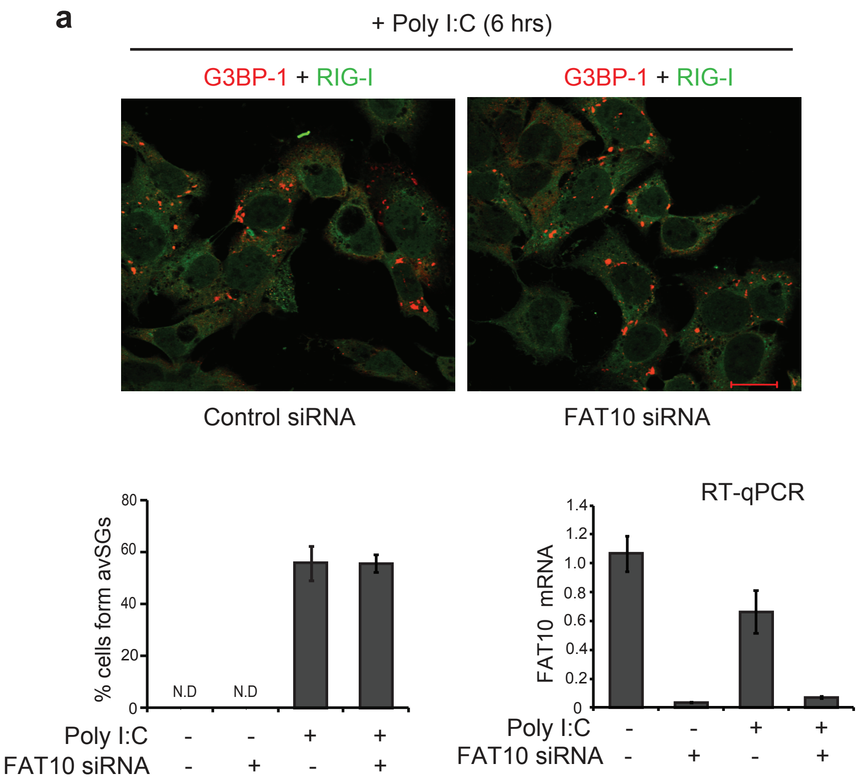

Tables 1. siRNA and primers information

siRNA information

h TRIM25 siRNA	sense: 5’-CCAUAGACCUCAAAAACGATT-3’<br>antisense: 5’-UCGUUUUUGAGGUCUAUGGTG-3’
Negative control	sense: 5’-UUCUCCGAACGUGUCACGUTT-3’<br>antisense: 5’-ACGUGACACGUUCGGAGAATT-3’
h FAT10 siRNA	sense: 5’-GAGACUAAGACGGGUAUAATT-3’<br>antisense: 5’-UUAUACCCGUCUUAGUCUCTT-3’
Negative control	sense: 5’-UUCUCCGAACGUGUCACGUTT-3’<br>antisense: 5’-ACGUGAACACGUUCGGAGAATT-3’

Primers for RT-qPCR

h FAT10 primers	Forward: 5’- CCCAATGCTTCCTGCCTC -3’<br>Reverse: 5’- TTTCAGGGTAAGGTGGATGG -3’
h TRIM25 primers	Forward: 5’- AGTGGTTCAACACCAAGAT -3’<br>Reverse: 5’- GGAGCAGATGGAGAGTGT -3’
GAPDH primers	Forward: 5’-AGGTGAAGGTCGGAGTCAAC -3’<br>Reverse: 5’- TGGAAGATGGTGATGGGATT-3’

Supplementary Figure legends

Supplementary Fig. 1 ***a*****,** HCT116 cells were mock-treated (untreated) or infected with influenza A/PR/8/34 DelNS1 (IAV DelNS1) for 9 h. Cells were fixed and stained with α-RIG-I and α-G3BP1 antibodies. ***b***, HepG2 cells transfected with Flag-FAT10 or Flag-CMV (empty vector), and poly I:C treated for 6 h before fixation. Cells were stained with α-Flag, α-RIG-I and α-G3BP1 antibodies.

Supplementary Fig. 2 ***a*****,** HEK293FT cells were transfected with indicated plasmids for 48 h. Cells were treated with 200 μM Chloroquin for 6 h before lysis. Mock means untreated. ***b*****,** HEK293FT cells were transfected with indicated plasmids for 48 h. After harvesting, the soluble and insoluble fractions were prepared using Passive lysis buffer. The protein levels of FAT10 and CARD in each fraction were analyzed. ***c*****,** Transfected HEK293FT cells (10%) were fractionated into RIPA soluble and RIPA insoluble, and the remaining cells (90%) were separated into cytosol and mitochondria fraction. Protein levels of FAT10 and CARD in each fraction were analyzed. ***d*****,** HEK293FT cells were transfected with indicated plasmids, and the protein levels of FAT10 and CARD in the cytosol and mitochondria enriched fraction were examined.

Supplementary Fig. 3 ***a*****,** HepG2 cell was transfected with FAT10 siRNA or control siRNA for 42 h, followed by stimulation with Poly I:C (10 μg/ml) for 6 h. Cells were fixed and stained with α-RIG-I and α-G3BP-1 antibody**.** In each sample, % cells with stress granules were calculated from about 100 cells (10 random fields, each field containing 8-12 cells). Data presented in graph was obtained from three independent samples, mean ± SD (error bar). N.D means no detection. At the same time, RT-qPCR was performed to examine FAT10 mRNA level.
